# The gut microbiome of wild American marten in the Upper Peninsula of Michigan

**DOI:** 10.1371/journal.pone.0275850

**Published:** 2022-11-03

**Authors:** Diana J. R. Lafferty, Erin A. McKenney, Sierra J. Gillman, Chris D. Kailing, Myles C. Walimaa, Macy J. Kailing, Brian J. Roell

**Affiliations:** 1 Department of Biology, Wildlife Ecology and Conservation Science Lab, Northern Michigan University, Marquette, Michigan, United States of America; 2 Department of Applied Ecology, North Carolina State University, Raleigh, North Carolina, United States of America; 3 School of Environment and Forest Sciences, University of Washington, Seattle, Washington, United States of America; 4 Department of Earth, Environmental, and Geographical Sciences, Wildlife Ecology and Conservation Science Lab, Northern Michigan University, Marquette, Michigan, United States of America; 5 Department of Biological Sciences, Virginia Polytechnic Institute and State University, Blacksburg, Virginia, United States of America; 6 Institute for Critical Technology and Applied Science, Virginia Polytechnic Institute and State University, Blacksburg, Virginia, United States of America; 7 Michigan Department of Natural Resources, Marquette, Michigan, United States of America; Universidad Austral de Chile, CHILE

## Abstract

Carnivores are ecologically important and sensitive to habitat loss and anthropogenic disruption. Here we measured trophic level and gut bacterial composition as proxies of carnivore ecological status across the Upper Peninsula, Michigan, for wild American marten (*Martes americana*; hereafter marten). In contrast to studies that have focused on omnivorous and herbivorous species, we find that marten, like other carnivore species without a cecum, are dominated by Firmicutes (52.35%) and Proteobacteria (45.31%) but lack Bacteroidetes. Additionally, a majority of the 12 major bacterial genera (occurring at ≥1%) are known hydrogen producers, suggesting these taxa may contribute to host energy requirements through fermentative production of acetate. Our study suggests that live trapping and harvest methods yield similar marten gut microbiome data. In addition, preserving undisturbed forest likely impacts marten ecology by measurably increasing marten trophic level and altering the gut microbiome. Our study underscores the utility of the gut microbiome as a tool to monitor the ecological status of wild carnivore populations.

## Introduction

Human-mediated environmental changes can influence the evolution and ecology of diverse wildlife [1–3]. Among the 5,498 described mammal species [4], terrestrial carnivores are among the most threatened on Earth [5], with many populations suffering rapid population declines and substantial range declines [6, 7]. Factors contributing to carnivore population declines are often linked to expanding human populations [8] and subsequent anthropogenic activities, such as deforestation [9, 10], large-scale agricultural development [4, 11], wildlife overexploitation [12], competition with invasive species introduced by humans [13], and prey depletion caused by human hunters [14]. However, while the external threats that carnivores face from their degraded environment are relatively well-known (e.g., deforestation, overexploitation), the consequences of those external threats on the carnivores’ internal environments are almost entirely unknown, specifically changes in their gut microbiota.

Over the past decade, mammalian microbiome research has provided a suite of integrated tools with exceptional potential to advance our understanding of mammalian ecology and evolution [15–18], thereby improving the conservation of diverse species [17, 19]. Mammals provide a diverse array of habitats for microorganisms to populate (e.g., ears, nose, between the toes), yet the majority of mammalian-associated microbial communities inhabit the gastrointestinal tract and perform vital metabolic functions (e.g., facilitate energy uptake, modulate immune response, trigger tissue development, synthesize vitamins) [20–24]. For example, recent evidence suggests that increased gut microbial diversity can increase host resistance to parasites [25, 26]. Thus, integrating gut microbiome assessments into ongoing population monitoring initiatives may provide new perspectives regarding the status and potentially the health of wild carnivore populations.

Several factors influence the composition of gut microbes in mammals, including host phylogeny [27, 28], life stage [29], and diet [16, 30, 31]. In addition, mammalian gut microbiomes are sensitive to habitat perturbations [17, 32, 33]. For example, forest disturbances that change the quality or availability of food resources may force a dietary shift that alters a host’s gut microbiota, which can lead to dysbiosis [17, 33, 34]. Amato et al. 2013 found that herbivorous black howler monkeys (*Alouatta pigra*) inhabiting disturbed forests had reduced gut microbial diversity compared to monkeys inhabiting undisturbed forests, and suggested they may suffer negative health outcomes resulting from microbiome depletion. Carnivores have generally evolved shorter guts compared to omnivorous or herbivorous species and may thus be more vulnerable to environmental drivers of gut microbial dysbiosis, as faster passage rates leave hosts less time to attenuate microbial membership. The vast majority of mammalian gut microbiome research has been conducted in controlled laboratory settings on model organisms (e.g., rodents, non-human primates) or has focused on connections to human health [15], and even fewer studies have focused on carnivores. However, gut microbiome analyses may offer valuable insights into carnivore health, nutrition, behavior, life history, and disease dynamics. Understanding how carnivore gut microbiomes are influenced by macro-ecological processes will deepen our understanding of their ecology and evolution, with substantial potential for informing carnivore conservation and habitat assessments.

While the gut microbiomes of other mustelid species have received limited attention, the American marten (*Martes americana;* hereafter marten) gut microbiome has yet to be characterized. Although marten are classified as a species of least concern by the IUCN [4], marten are limited to conifer-dominated forests [35–37] and several populations are considered highly vulnerable to disturbance across large portions of their range in the United States (e.g., State Endangered Species in Wisconsin, Vermont). Further, marten are recognized as a culturally and ecologically important species [38] and a furbearer of historic economic value prior to overexploitation across much of North America [39]. Marten forage across trophic levels, feeding on rodents, lagomorphs, birds, and invertebrates, as well as fruit (e.g., *Vaccinium* spp.), carrion, and human foods when available [40]. Marten diets may vary across the landscape as a result of differential access to food resources and variable human disturbance. Their conservation significance in combination with their dietary responses to changing environments make the marten an excellent model for investigating the effects of human-mediated forest disturbance on carnivore gut microbiomes. The goals of this study were therefore to (a) characterize the gut bacterial diversity of an obligate forest carnivore, (b) assess variation in trophic levels across varying levels of human influence on the landscape, and (c) investigate the relationships among these factors.

We hypothesized that marten trophic position would vary relative to human impacts. We also hypothesized that gut bacterial community structure would vary across a gradient of human impacts given the relationships between forest disturbance, diet, and gut microbiota. We predicted that trophic level would correlate inversely with disturbance, with marten occupying a higher trophic position in areas with lower human impacts because undisturbed forests may host more robust food webs compared to disturbed forests. We also predicted that marten in disturbed forest may host greater bacterial alpha diversity (to facilitate the digestion of more omnivorous diets) and greater bacterial beta diversity (reflective of landscape heterogeneity) compared to marten in undisturbed habitat, because marten may supplement a carnivorous diet with more vegetation (e.g., *Rubus* spp. [raspberries]) in disturbed areas. Thus, we also investigated whether marten gut microbiomes can serve as an indicator of resource quality and availability, providing a novel and noninvasive tool for monitoring population health across increasingly human-impacted landscapes.

## Methods

### Study area

**Fig 1:**
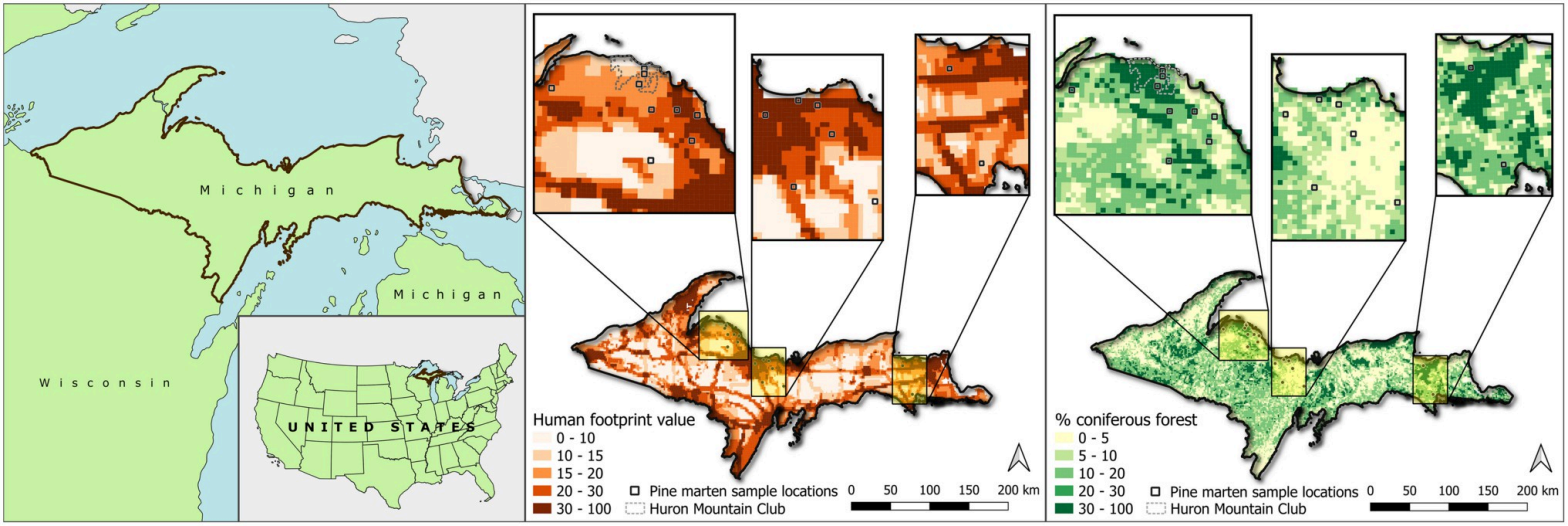
Map of the Upper Peninsula of Michigan with an inset of the United States for geographic reference (left). Upper Peninsula of Michigan showing human footprint value across the Upper Peninsula with call-outs showing the Human Footprint value for marten sample locations (middle). Coniferous forest cover for marten sample locations (right). Maps created using QGIS 3.10.12 A Coruña. Michigan boundary, Great Lakes, and Public Land Survey System shapefiles from State of Michigan (https://gis-michigan.opendata.arcgis.com). United States boundaries shapefile from US Census Bureau (https://catalog.data.gov). Huron Mountain Club shapefile from Huron Mountain Club. Shapefiles and rasters processed using R (version 4.0.3), R Studio (version 1.3.1093), *tidyverse* (version 1.3.1), *raster* (version 3.5.2), *sf* (version 1.0.3).

Marten were sampled from across the Upper Peninsula (UP) of Michigan (MI), USA (47°00’–45°09’N, 90°18’–84°37’W; Fig 1) from December 2018 through March 2019. Elevation across the UP ranges between approximately 170 m to 600 m above sea level and temperatures varied from a low of -16°C to a high of 3°C during the sampling period. Land cover across the UP is diverse, consisting of deciduous forests, conifer forests, mixed deciduous-conifer forests, swamps, meadows, and an extensive shoreline along Lake Superior to the north and Lake Michigan and Huron to the south. The region has a long history of timber extraction resulting in forests of various successional stages. The Huron Mountain Club is a privately owned 8,000 ha system that includes primarily hemlock (*Tsuga canadensis*) forest and mature mesic coniferous forest with minimal human impact; we therefore putatively classified Huron Mountain Club property as “undisturbed forest” and all non-Huron Mountain Club samples as coming from “disturbed forest”.

### Sample collection

We collected marten fecal samples opportunistically from animals either legally harvested by trappers (*n* = 16), or live-captured and released (*n* = 5) during the winter spanning November 2018-March 2019. Legally harvested animals remained frozen at capture until they were brought to the Michigan Department of Natural Resources (MDNR) office in Marquette, MI by the individual that harvested the animal (per state guidelines). Individuals granted permission to MDNR personnel to collect approximately 10 hairs from the base of the tail as well as gastrointestinal tract samples from the colon using a sterile wooden tongue depressor. We placed hair samples in a coin-envelope and colon content samples in a sterile Eppendorf tube containing 95% ethanol. Both sample types were stored at room temperature [41].

We randomly selected live-trapping locations at Huron Mountain Club (HMC) and public lands in Ishpeming, MI in ArcGIS Pro [42] using a 3km^2^ × 3km^2^ grid overlay on each sampling area, which is approximately the size of female marten home ranges [36], and a random number generator to identify grid cells for sampling. We set a live-trap within each randomly selected grid cell at locations with observed marten sign (e.g., tracks) or in structurally complex areas with preferred cover (i.e., large trees and high amounts of deadfall) to maximize capture success. We trapped marten using custom waterproof wooden box traps (60cm × 30cm × 20cm) designed for cold weather conditions and lined with straw bedding for insulation. Traps were baited with chicken, deer, beaver, or pork and lured with Gusto, Tree Climber, Skunk Junk (Pennock, MN), or Lenon’s Fox #3 Nature’s Call (Turner, MI). We selected baits and lures based on conversations with local marten trappers. We applied lures to a stick approximately one meter in height positioned next to the trap and checked traps every ~12 hours. Upon successful capture, we immediately released animals from the trap and searched for hair and feces. We placed hair samples in pre-labeled coin envelopes. Fecal samples were placed in a sterile Eppendorf 15 mL tube containing 7 mL of 95% ethanol using a sterile tongue depressor. All samples were stored at room temperature until processing. We sterilized and replaced straw bedding in the reset trap after each capture.

All live-capture procedures were approved by Northern Michigan University Institutional Animal Care and Use Committee (IACUC) and we confirm that all procedures were performed in accordance with approved protocol #327. For harvested specimens, we received an exemption from review from the NMU IACUC committee because samples were collected from dead marten that were legally harvested by individuals who were not involved with this research. All samples were collected under a Michigan DNR-Wildlife Division-Scientific Collector’s Permit (#SC 1613).

### Human footprint score and land cover classification

To evaluate the potential influence of human landscape disturbance on marten microbiomes, we calculated the Human Footprint Score (HFS) [43], which provides a measure of direct human influence on terrestrial ecosystems using data on human settlement, recreational access, landscape transformation, and electrical power infrastructure [44–46]. While we obtained precise sample collection locations from live-captured/released animals, samples opportunistically obtained from trappers were reported at the “section” level of the United States Public Land Survey System (US PLSS) [47]; we therefore calculated the HFS at the US PLSS section level for all samples (Fig 1). Using the *raster* [48] and *sf* [49] packages in Rstudio [50], values from the HFS were extracted from each US PLSS section and the mean was calculated.

We used the LANDFIRE Existing Vegetation Types 1.4.0 (EVT) [51] dataset for classifying land cover at the section level. The EVT values were extracted for each US PLSS section and the “EVT_PHYS” attribute was used to calculate the relative percentage of coniferous forest for each US PLSS section in which each sample was obtained (Fig 1).

### DNA extraction for 16S rRNA amplicon sequencing

We extracted DNA from ~0.25g of each marten fecal sample using the DNeasy PowerSoil Kit (QIAGEN, Hilden, Germany), following the manufacturer’s protocol with the addition of an initial heat-step increased to 10 minutes at 65°C and a second final elution [29]. We assessed the quality and quantity of DNA yields via spectrophotometric measurements using a NanoDrop 2000c (ThermoFischer Scientific, Massachusetts, USA). All samples were aliquoted in equimolar ratios and sent to Argonne National Laboratory (Lemont, IL, USA) for PCR amplification of the V4 region of the 16s rRNA gene and paired-end DNA sequencing. We targeted the v4 gene region the 16S rRNA gene using the forward primer 515F (5′-GTGCCAGCMGCCGCGGTAA-3′) and the reverse primer 806R (5′-GGACTACHVHHHTWTCTAAT-3′) and 2 × 150 paired-end reads on Illumina’s MiSeq platform. As standard laboratory protocol, Argonne National Laboratory includes negative PCR controls in every plate amplified and proceeds with pooling and sequencing if the negative controls are clean.

### Bioinformatic analysis

We imported Multiplexed EMP-paired-end sequence reads into Quantitative Insights Into Microbial Ecology (QIIME2), version 2020.8 [52]. We then demultiplexed, joined, denoised and truncated all sequences to 150 bp, subsequently removing chimeras and residual Phix reads and dereplicating sequences. We called amplicon sequence variants (ASVs) using the DADA2 QIIME2 plugin [53]. We used the SILVA 99 database version 138 for the V4 region [54] to assign taxonomic classification within QIIME2 using a trained Naïve Bayes sklearn classifier to classify organisms at the genus level [55]. Sequences were aligned with the MAFFT plugin for phylogenetic diversity analysis [56], which removes highly variable positions in the process. Samples were further filtered to remove chloroplast, mitochondria, and unassigned sequences. Prior to rarefaction sequences had a mean depth of 40,070 ± 2372^2^ SE. After filtering, all samples were retained (*n* = 21) with sequenced depth ≥ 15,900.

### Stable isotope sample preparation and analysis

Ratios of heavy to light naturally occurring stable isotopes (e.g., carbon: ^13^C/^12^C [δ^13^C]; nitrogen: ^15^N/^14^N [δ^15^N]) in animal tissues (e.g., hair, claws, muscle, blood) can be used to investigate individual trophic positions within a food web [57–59]. For example, nitrogen fractionation of 3–4 ‰ (parts per mil) occur with each trophic level [60], such that as the trophic level of a food resource increases, the δ^15^N values of the consumer’s tissues increases [57, 61], whereas δ^13^C values exhibit little fractionation across trophic levels, thereby reflecting basal food resources use (e.g., plant consumption) [62]. As such, we removed hair follicles from whole hair samples from 14 individuals (11 harvested; 3 live trapped) and sent these samples to Cornell University Stable Isotope Laboratory for standard stable carbon and nitrogen isotope analysis using a Thermo Delta V isotope ratio mass spectrometer interfaced to a NC2500 elemental analyzer. We report isotopic values in delta (δ) notation such that δ^13^C or δ^15^N = [(R_sample_/R_standard_)– 1] x 1000, where R_sample_ and R_standard_ are the ^13^C/^12^C or ^15^N/^14^N ratios of the sample and standard, respectively. The standards are PeeDee Belemnite limestone for carbon and atmospheric N_2_ for nitrogen.

### Statistical analysis

We used *qiime2R* (version 0.99.12) [63] to import QIIME2 artifacts into Rstudio (version 1.2.5003) [64] for statistical analysis using R (version 3.6.2) [50]. Samples were rarefied to 15,900 sequences/sample (mean: 40,070; range: 15,908–56,345). We calculated the nonparametric Chao1 estimator of abundance-based species richness, which calculates the expected number of ASVs based on observed ASVs [65, 66], and Shannon diversity, which calculates the proportion of ASV *I* relative to the total number of ASVs in the community, with the alpha function in the *microbiome* package (version 1.6.0) [67]. We calculated Faith’s phylogenetic diversity (PD), which calculates the total branch lengths on a phylogenetic tree of all members in the microbiome community [68], with the pd function in the *picante* package (version 1.5) [69]. We used Wilcoxon rank sum pairwise comparisons (hereafter reported as W) to test for significant differences between harvested and live-trapped marten. We calculated weighted and unweighted UniFrac distances, which quantify the shared phylogenetic diversity between pairs of microbial communities and incorporate either species abundance or presence/absence, respectively [70, 71], with the distance function in the *phyloseq* package (version 1.28.0) [72].

We first compared live-trapped versus harvested individuals, to determine whether their respective gut bacterial communities were sufficiently similar to be considered a single population. Specifically, we used perMANOVA to compare community composition and Mann Whitney U tests to determine whether alpha diversity values differed significantly between live-trapped versus harvested marten microbiomes. To investigate the relationships among habitat, trophic position, and gut bacterial alpha diversity we used regression models in which the alpha diversity indices and δ^15^N were modeled separately as a function of mean HFS and percent conifer land cover. Both HFS and percent land cover were centered and scaled using the scale function in base R, and we checked residuals to confirm model requirements (e.g., normality, homoscedasticity, residuals). We used δ^15^N from whole hairs to estimate trophic position for all animals from which hair samples were obtained (*n* = 14; 11 harvested, 3 live-trapped), and Faith’s PD was log-transformed prior to analysis. To understand the impact of mean HFS and percent conifer cover on gut bacterial variation, we compared pairwise Euclidean distance matrices with UniFrac beta diversity distances using Mantel tests [73]. Mantel tests were based on the Pearson correlation in the *vegan* package (version 2.5–6) [74].

## Results

### Characterization of the marten microbiome

**Fig 2:**
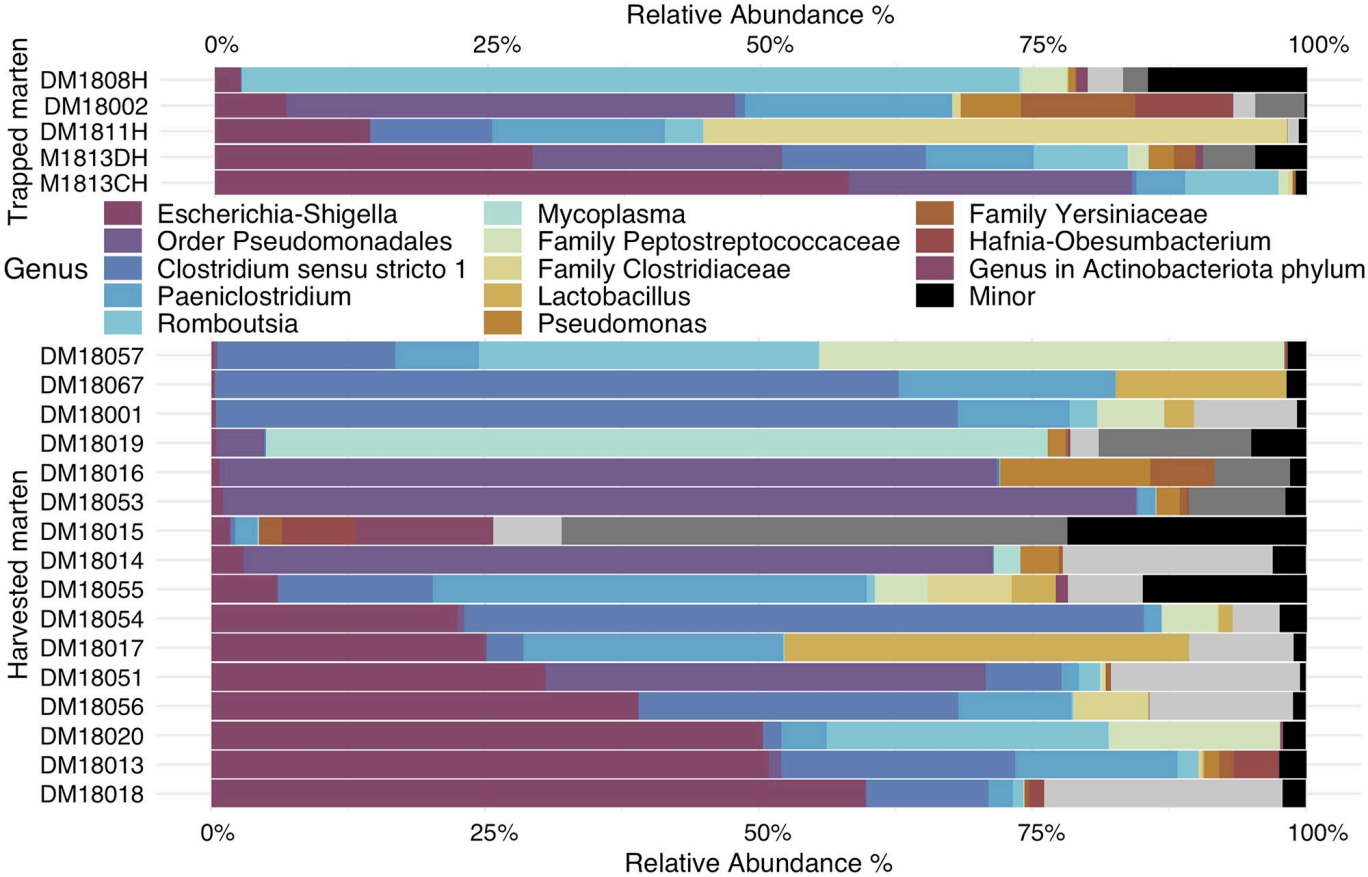
Bacterial community composition for live-trapped (n = 5) and harvested marten (n = 16) (*Martes americana*) from the Upper Peninsula of Michigan.

We first compared the bacterial community composition between harvested and live-trapped marten (Fig 2). Firmicutes were the most abundant phylum in harvested marten (53.47% ± 8.48%) and the second most abundant in live-trapped marten (48.76% ± 15.24%), whereas Proteobacteria was the most abundant phylum in live-trapped marten (49.76% ± 15.81%) and the second most abundant in harvested marten (43.90% ± 8.23%; Table 1). Additionally, Actinobacteriota were the third most abundant phylum in harvested marten, but no other phylum was present at >1% abundance in live-trapped marten. At the genus level, harvested marten harbored 12 major genera and live-trapped marten harbored 11 major genera (Table 1). While harvested and live-trapped marten shared seven major genera, harvested marten harbored four unique major genera in the phylum Firmicutes: *Mycoplasma*, *Romboustsia*, *Carnobacterium*, *Terrisporobacter*. In contrast, live-trapped marten harbored four unique genera in the phylum Proteobacteria: *Pseudomonas*, an unknown genus in the family Yersiniaceae, *Hafnia-Obesumbacterium*, *Sphingomonas*, and an unknown genus in the order Enterobacterales. Despite these differences in presence or absence of genus-level membership, we did not detect statistically significant differences in either alpha diversity (Chao1: W = 57, p = 0.53; Shannon: W = 44, p = 0.80; PD: W = 46, p = 0.91) or community composition (perMANOVA: weighted UniFrac: F = 0.25, p = 0.78, [homogeneity of variance: F = 1.8, p = 0.68] between live-trapped and harvested individuals. However, when considering only species presence/absence, we did detect differences between live-trapped and harvested individuals (unweighted UniFrac: F = 1.73, p = 0.02, [homogeneity of variance: F = 1.55, p = 0.24]), which was unsurprising because unweighted UniFrac disproportionately weights taxa at low relative abundances. We therefore characterize all marten samples below as a single population.

**Table 1:** Mean relative abundance of major (≥1%) bacterial phyla and genera in the fecal samples of harvest and live-trapped American marten (*Martes americana*; n = 21).

		Harvested n = 16		Live-trapped n = 5	
Phylum	Genus	Abundance	SD	Abundance	SD
**Firmicutes**	Clostridium_sensu_stricto_1	18.61%	24.19%	5.10%	6.05%
	Paeniclostridium	8.64%	10.93%	9.86%	7.96%
	Mycoplasma	4.63%	17.86%	< 1%	NA
	Ambiguous_taxa	4.43%	10.60%	10.81%	23.96%
	Romboutsia	4.10%	9.70%	< 1%	NA
	Lactobacillus	3.81%	9.76%	< 1%	NA
	Carnobacterium	1.09%	3.76%	< 1%	NA
	Terrisporobacter	1.04%	2.16%	< 1%	NA
	Minor genera (total)	7.10%	NA	4.81%	NA
	**Total Firmicutes**	**53.46%**	**8.48%**	**48.76%**	**15.24%**
**Proteobacteria**	Escherichia-Shigella	18.27%	21.62%	22.26%	22.73%
	Order_ Pseudomonadales	16.83%	30.23%	17.79%	17.61%
	Pseudomonas	1.38%	3.41%	1.69%	2.26%
	Family Yersiniaceae	< 1%	NA	2.53%	4.49%
	Hafnia-Obesumbacterium	< 1%	NA	1.82%	4.08%
	Sphingomonas	< 1%	NA	1.06%	1.41%
	Order_ Enterobacterales	< 1%	NA	1.00%	0.83%
	Minor genera (total)	7.42%	NA	1.65%	NA
	**Total Proteobacteria**	**43.90%**	**8.23%**	**49.80%**	**15.81%**
**Actinobacteriota**	All minor taxa	1.53%	1.16%	< 1%	NA
**Total Minor Phylum**	All minor taxa	1.10%	NA	1.44%	NA

The total sampled marten gut bacterial communities for the current study comprised 22 phyla, three of which were found at relative abundance of ≥1%: Firmicutes (52.36% ± 33.27% SD), Proteobacteria (45.31% ± 32.7% SD), and Actinobacteriota (1.41% ± 4.1% SD; Table 2). At the genus level, 419 genera were identified, 12 of which occurred at relative abundance of ≥1%: *Escherichia-Shigella* (19.15% ± 21.33% SD), an unknown genus in the Order Pseudomonadales (17.14% ± 27.41% SD), *Clostridium sensu stricto 1* (15.39% ± 21.94% SD), *Paeniclostridium* (8.98% ± 10.21% SD), *Romboutsia* (7.48% ± 16.89% SD), *Mycoplasma* (3.55% ± 15.55% SD), and unknown genera in the Family Peptostreptococcaceae (3.52% ± 9.42% SD), *Lactobacillus* (2.91% ± 8.54% SD), unknown genus in the Order Clostridiaceae (2.91% ± 11.70% SD), *Pseudomonas* (1.47% ± 3.17% SD), an unknown genus in the Family Yersiniaceae (1.17% ± 2.52% SD), and *Hafnia-Obesumbacterium* (1.03% ± 2.47% SD; Table 1).

**Table 2:** Mean relative abundance of major (≥ 1%) bacterial phyla and genera in the fecal samples of American marten (*Martes americana; n* = 21) sampled in the Upper Peninsula of Michigan.

Phylum	Genus	Abundance	SD
**Firmicutes**	*Clostridium_sensu_stricto_1*	15.39%	21.94%
	*Paeniclostridium*	8.98%	10.21%
	*Romboutsia*	7.48%	16.89%
	*Mycoplasma*	3.55%	15.55%
	Unknown genus: Family Peptostreptococcaceae	3.52%	9.42%
	*Lactobacillus*	2.91%	8.54%
	Unknown genus: Order Clostridiaceae	2.91%	11.70%
	Minor genera (total)	7.61%	NA
	**Total Firmicutes**	**52.35%**	**33.16%**
**Proteobacteria**	Escherichia-Shigella	19.15%	21.33%
	Order_Pseudomonadales	17.14%	27.41%
	Pseudomonas	1.47%	3.17%
	Family_Yersiniaceae	1.17%	2.52%
	Hafnia-Obesumbacterium	1.03%	2.47%
	Minor genera (total)	5.35%	NA
	**Total Proteobacteria**	**45.31%**	**32.70%**
**Actinobacteriota**	All minor taxa	1.40%	4.10%
**Total Minor Phyla**	All minor taxa	0.94%	NA

### The influence of HFS/conifer cover on trophic position

While bacterial taxa occurred at different levels of relative abundance in live-trapped and harvested marten alpha diversity did not differ significantly between the two groups (Table 3). We did, however, identify differences in environmental metrics. For example, live-trapped and harvested marten exhibited different isotopic signatures. In addition, live-trapped marten were associated with greater conifer land cover (Table 3). Because live-trapped and harvested marten bacterial communities did not differ, we combined samples from both groups for additional analyses.

**Table 3:** Descriptive statistics associated with environmental covariates (Human Footprint Score [HFS], % conifer cover), trophic position (nitrogen [δ^15^N] and carbon [δ^13^C] stable isotope values), and alpha diversity indices (Faith’s PD, Shannon, Chao1) for live-trapped (n = 5) and harvested (n = 16) American marten (*Martes americana*) sampled in the Upper Peninsula of Michigan during the 2018–2019 marten harvest season.

Harvested n = 16				Live-trapped n = 5		
	Mean ± SD	Median	Range	Mean ± SD	Median	Range
**HFS**	27.91 ± 12.13	25.67	10–56	13.35 ± 3.40	14	9–16
**% Conifer**	0.12 ± 0.12	0.06	0.01–0.49	0.36 ± 0.01	0.36	0.35–0.38
**δ** ^**15**^ **N**	5.77 ± 0.719	5.76	4.75–7.14	7.29 ± 1.36	7.71	5.77–8.38
**δ** ^**13**^ **C**	-22.32 ± 0.34	-22.30	-22.90 –-21.61	-19.86 ± 2.39	-18.63	-2.61 –-18.33
**Faith’s PD**	6.94 ± 8.03	3.73	2.15–30.49	7.16 ± 5.57	4.63	2.40–16.35
**Shannon**	1.86 ± 0.89	1.66	0.79–4.74	1.93 ± 0.51	2.03	1.24–2.44
**Chao1**	99.66 ± 161.74	43.5	22.00–648.65	79.44 ± 81.15	36.35	27.50–218.78

**Fig 3:**
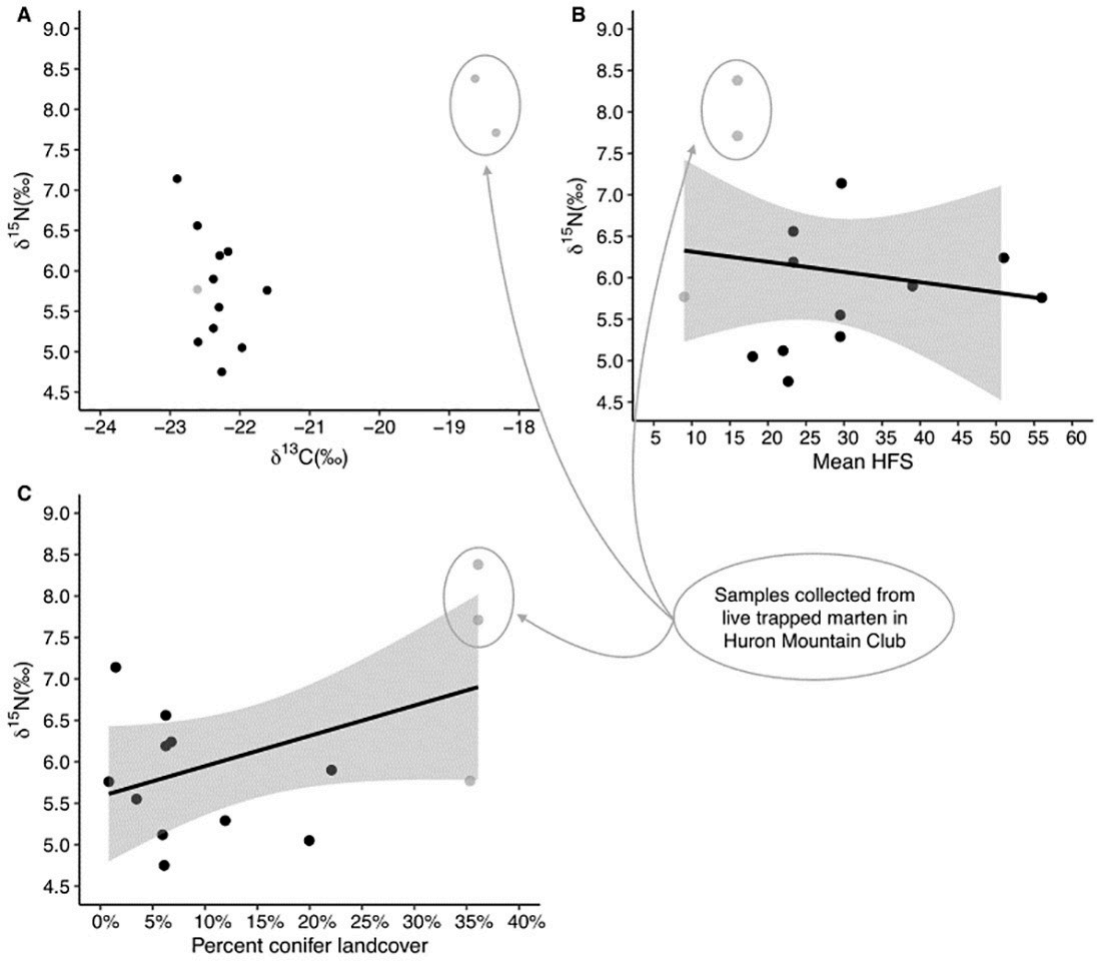
Trophic position (δ^15^N) of American marten (*Martes americana*) harvested (n = 11; black) or live trapped (n = 3; grey) in the Upper Peninsula of Michigan across sites with different (A) distribution of carbon and nitrogen stable isotope ratios, (B) Human Footprint Scores (HFS), and (C) percent conifer landcover. The two circled samples were collected from live-trapped marten located on the Huron Mountain Club property, a privately owned 8,000 ha system that includes old growth/primary hemlock (*Tsuga canadensis*) forest and mature mesic coniferous forest with minimal human impact.

For all marten sampled, mean HFS ranged from 9–56 and percent conifer land cover ranged from 0.8% to 36.1% (Table 4). Stable isotope values ranged from -18.33‰ to -22.90‰ for δ^13^C and from 4.75‰ to 8.38‰ for δ^15^N, with two marten from HMC displaying the highest trophic position and enriched δ^13^C (Fig 3A). Although marten from undisturbed habitat had the highest trophic position, neither mean HFS nor percent conifer land cover were strong predictors for trophic position (HFS: Estimate coef = 0.16, t-value = 0.51, 95% Confidence Intervals [CI] = -0.53 to 0.86; % Conifer: Estimate coef = 0.65, t-value = 1.73, CI = -0.18 to 1.49). In both comparisons, however, the two samples from undisturbed habitat (HMC3, HMC4) were isotopically distinct from the other samples (Fig 3B and 3C).

**Table 4:** Descriptive statistics associated with environmental covariates (Human Footprint Score [HFS], % conifer cover), trophic position (nitrogen [δ^15^N] and carbon [δ^13^C] stable isotope values), and alpha diversity indices (Faith’s PD, Shannon, Chhao1) for American marten (*Martes americana*; n = 21) sampled in the Upper Peninsula of Michigan.

All marten n = 21			
	Mean ± SD	Median	Range
**HFS**	24.98 ± 12.42	23	9.00–56.00
**% Conifer**	0.17 ± 0.15	0.09	0.01–0.49
**δ** ^**15**^ **N**	6.10 ± 1.05	5.84	4.75–8.38
**δ** ^**13**^ **C**	-21.79 ± 1.44	-22.30	-22.90 –-18.33
**Faith’s PD**	6.53 ± 7.27	4.03	2.15–30.49
**Shannon**	1.86 ± 0.82	1.66	0.79–4.74
**Chao1**	88.65 ± 145.85	38.63.5	22.00–648.65

### The influence of HFS/conifer cover on alpha and beta diversity

**Fig 4:**
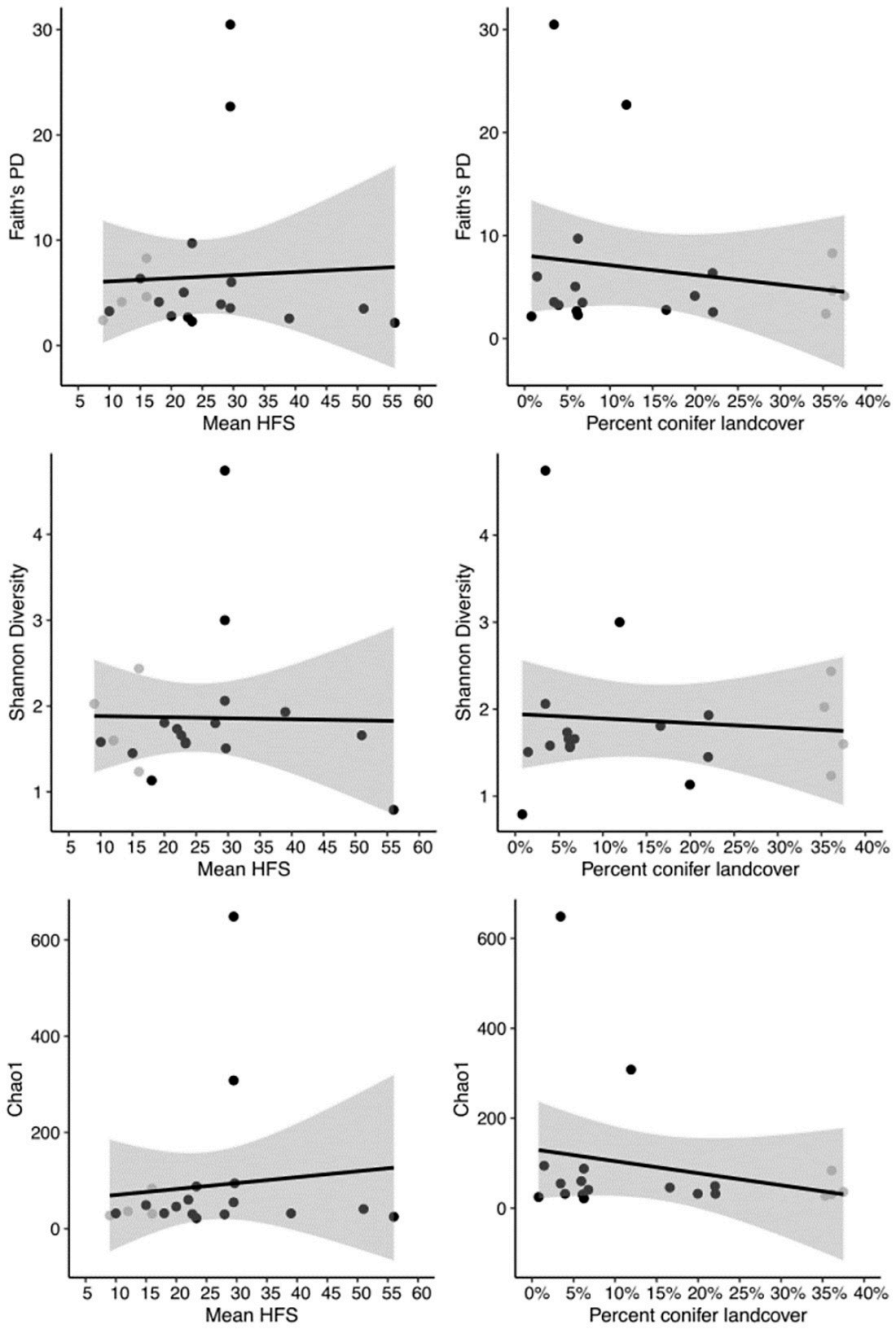
Individual alpha diversity scores for American marten (*Martes americana*) harvested (black) or live-trapped (grey) across sites in the Upper Peninsula of Michigan with different Human Footprint Scores (HFS) and percent conifer land cover.

We found no strong relationships between bacterial alpha diversity indices and either mean HFS, percent conifer land cover, or δ^15^N (Table 5; Fig 4). Mantel tests revealed that mean HFS and percent conifer cover did not significantly influence gut bacterial community composition (weighted UniFrac, r = -0.13, *p* = 0.82; unweighted UniFrac, r = -0.13, *p* = 0.86).

**Table 5:** Regression estimates predicting gut bacterial alpha diversity indices in American marten (*Martes americana*).

Faith’s PD	Estimate (SE)	Lower 95% CI	Upper 95% CI
(Intercept)	1.55 (0.17)	2.97	10.08
Scaled mean HFS	-0.09 (0.19)	-4.29	3.78
Scaled % conifer cover	-0.11 (0.19)	-5.50	2.58
Shannon Diversity	Estimate (SE)	Lower 95% CI	Upper 95% CI
(Intercept)	1.86 (0.19)	1.46	2.27
Scaled mean HFS	-0.05 (0.22)	-0.52	0.41
Scaled % conifer cover	-0.09 (0.22)	-0.55	0.38
Chao1	Estimate (SE)	Lower 95% CI	Upper 95% CI
(Intercept)	88.65 (33.38)	18.22	159.08
Scaled Mean HFS	-0.42 (37.91)	-80.40	79.56
Scaled % conifer cover	-36.68 (37.91)	-116.66	43.30

## Discussion

Here we present the first characterization of the gut microbiome in wild marten. In contrast to most mammalian gut microbiome studies, which have generally focused on omnivorous and herbivorous species with ceca, and similar to the findings of a study of the gut microbiome of North American river otters [75], the marten gut community is dominated by Firmicutes (52.35%) and Proteobacteria (45.31%). Further, the marten gut microbiome comprises only 12 major genera (Table 2) that tend to be generalist bacteria previously associated with early stages of succession in the gastrointestinal tract (*Clostridium sensu stricto 1* and *Lactobacillus*; [76]) or disease (*Clostridium sensu stricto 1* [77]; *Escherichia-Shigella* [78]; *Mycoplasma* [79]). We therefore propose that the marten’s rapid transit time may favor ruderal species that thrive in disturbed environments but preclude pathogenic effects, instead selecting for other specific functions that benefit the host. For example–*Clostridium sensu stricto 1*, *Peiniclostridium*, *Romboutsia*, *Escherichia-Shigella*, and *Halfnia-Obesumbacterium* are all key taxa in dark fermentative production of hydrogen gas [80, 81], a substrate used by many Firmicutes to produce acetate [82], which is in turn a primary energy source in peripheral host tissues where it is converted to ATP or adipose tissue [22]. We hypothesize that these genera, which induce disease states in humans and other species with prolonged gut transit times, can play adaptive roles in other host contexts such as carnivore guts. Microbial fermentation is considered a critical process for energy production in herbivores and omnivores [22] but has been generally overlooked or dismissed in carnivores. We therefore recommend that ecologists investigate the potential prevalence and importance of probiotic fermentation in carnivore species.

Live trapping versus harvest do not appear to bias bacterial data: both Chao1 and Faith’s PD estimates follow similar distribution patterns (Fig 3). The low alpha diversity values reported here likely indicate limited niche space related to high disturbance (i.e., rapid transit) rates in the marten gut–not to mention competition between hosts and their gut microbiomes for easily digested proteins and lipids. Chao1 is low (𝜇 = 88.65) but extremely variable (SD = 145.85) among individuals (Table 6), while Shannon diversity is consistently low (1.86 ± 0.82 SD) compared to values recorded for species with ceca (𝜇 ≅ 7 in woodrats [83], range = 3.2–7.7 in white-tailed deer [84], range ≅ 6–6.5 in lemurs [32]). McKenney et al. previously showed that alpha diversity increases with gut transit time [30] and confirmed that giant panda and red panda gut microbiomes are dominated by Firmicutes and Proteobacteria, but not Bacteroidetes. While more extensive sampling across carnivorous species is needed, we posit that this trend may extend to several wild carnivores studied to date, which suggests that species without a cecum may host more facultative anaerobes and bacterial taxa that are better adapted to metabolize protein and lipids compared to the anaerobic fiber-fermenting microbial specialists favored by omnivores and herbivores.

**Table 6:** Diversity and composition of gut microbial communities characterized in wild carnivore species that lack a cecum. We included the major taxa (comprising ≥1% relative abundance +/- SD, where available), as well as Bacteroidetes for comparison with omnivore/herbivore data. “Minor” indicates that the taxon was present, but at levels < 1% relative abundance; “NA” indicates that data were not available; “NP” indicates that a taxon was not present in the data.

Carnivore	American marten *Martes americana* (current study)	Sable *Martes zibellina* [89]	American black bear *Ursus americanus* [90]	Brown bear *Ursus arctos* [85]
Sample size	21	10	58	62
Sequencing Platform	Illumina MiSeq	Illumina MiSeq	Illumina MiSeq	Illumina MiSeq
Gene amplification region	16S v4	16S v3-v4	16S v4	16S v4
Primers	338F and 806R	338F and 806R	338F and 806R	338F and 806R
Reference database	Silva 99	Greengene	Silva 99	Silva 99
OTU vs. ASV method^1^	ASV^1^	OUT	ASV^1^	ASV^1^
Sequencing depth per sample	40,070 ± 2372^2^	47,405 ± 4872^2^	NA	NA
Normalization method, threshold	Rarefied to 15,900 seqs	Data were not normalized	SRS, C_min_ = 1,455	SRS, C_min_ = 4,087
Chao1	94.85 ± 144.68	516.3 ± 948.05	NA	166.33 ± 276.26
Shannon	1.88 ± 0.81	0.256 ± 0.15	1.72 ± 0.67	2.19 ± 1.16
Faith’s PD	6.99 ± 7.43	NA	3.99 ± 1.84	11.20 ± 10.51
Firmicutes	52.35± 33.16	38.23	60.26 ± 32.81	49.17 ± 16.52
Proteobacteria	45.31 ± 32.7	30.29	33.40 ± 29.97	32.30 ± 10.28
Actinobacteria	1.4 ± 4.1	28.15	Minor	2.11 ± 0.58
Epsilonbacteraeota	NP	NA	5.36 ± 10.39	7.74 ± 2.31
Fusobacteria	Minor	NA	Minor	Minor
Tenericutes	NP	NA	Minor	7.19 ± 1.93
Bacteroidetes	Minor	Minor	Minor	1.96 ± 0.91

^1^ ASV (Amplicon Sequence Unit) approach may result in greater estimated values of microbial taxonomic diversity compared to the OTU (Operational Taxonomic Unit) approach.

^2^ Values reported are mean ± SE.

Given the high level of among-individual variation detected in the bacterial communities of marten in this study, as well as the findings from other studies of carnivore gut microbiomes (e.g., mink [84], *Ursus* spp. [41, 85]), it is not surprising that we detected statistically significant differences in unweighted UniFrac between live-trapped and harvested marten. As a metric, unweighted UniFriac disproportionately weights taxa that are present at low relative abundances. It is likely that live trapped marten may experience greater levels of short-term stress compared to harvested animals. For instance, live-capture is known to trigger the hypothalamic–pituitary–adrenal (HPA) axis resulting in a measurable stress response (e.g., blood and/or hair cortisol concentration) [86] and a recent study of red squirrels (*Tamiasciurus hudsonicus*) found that bacterial diversity was lower in animals experiencing higher stress as indicated by higher levels of fecal glucocorticoid metabolites [87]. Further, live-capture often triggers defecation, ultimately increasing gut passage rate, which may increase the prevalence of Proteobacteria, a phylum that is ecologically more opportunistic and associated with disturbance and earlier successional stages as compared to Firmicutes, which dominated the bacterial community of harvested marten (Table 1). Another important consideration is that live-captured marten in our study were captured in relatively undisturbed forest on Huron Mountain Club property and occupied a higher trophic position (i.e., more carnivorous) compared to harvested marten in our study. While previous research shows that Proteobacteria are more abundant in domestic dogs and cats fed high-protein diets [88], additional studies are needed both to gauge the degree of among-individual variation with regards to unweighted UniFrac distances and to parse the effects of sampling methods (e.g., live-trapped versus harvested), extent of habitat disturbance, and trophic position on the gut microbiome.

Compared to the other carnivore species included in Table 6, marten are habitat specialists requiring conifer dominated forest. However, we did not detect a relationship between trophic level and HFS or percent conifer land cover (Fig 3B and 3C). Given the relatively small size of marten home-ranges (e.g., females 2.3 km^2^; ~8 km^2^ males) [36, 49] relative to the US PLSS section level, calculating land cover values at the US PLSS section level may not have provided high enough resolution to capture significant relationships. However, two marten sampled from Huron Mountain Club, which had the highest conifer land cover of the locations sampled (35%), held the highest trophic levels (δ^15^N = 8.39 and 7.71 and δ^13^C = -18.63 and -18.33, respectively, with all others falling between δ^15^N = 4.75–7.14 and δ^13^C = -21.61–22.90; Fig 1). While increasing the sample size could provide more meaningful ecological inference, perhaps marten inhabiting undisturbed habitat (e.g., primary/old growth forests) have different ecological relationships with their environment (e.g., more carnivorous) and therefore occupy detectably higher trophic positions compared to marten in disturbed habitat where berries (*Vaccinium* spp.) may be more abundant.

Recent studies have demonstrated that the gut microbiome can be used as an indicator of food resource use. For instance, black bear consuming processed anthropogenic foods host significantly degraded gut microbiomes [90], whereas brown bears with access to salmon host gut microbial communities that are distinguishable from populations limited to terrestrial food sources [85]. Given that marten consume a variety of prey across trophic levels, from terrestrial and aquatic environments and from both natural and anthropogenic sources, the gut microbiome of marten may be used to distinguish among animals consuming food items across a variety of sources and land covers with variable human influences. Indeed, landscape disruption has been shown to significantly affect the gut microbiome in howler monkeys [17] and red colobus monkeys [33]. In contrast, we did not detect strong relationships between marten gut microbiomes and the extent of forest disturbance in the current study. Our findings may reflect the marten’s comparatively simple gut morphology (i.e., lacking a cecum) and rapid gut passage rate from consumption to defecation. Rapid passage rates may preclude regulation by the immune system, and therefore may result in increased gut microbial variation among individuals–in addition to variation resulting from environmental and dietary perturbations [90]. Further, our sampling occurred during the winter when food resources are scarce. As such, sampling marten during months of greater resource availability (e.g., summer) might reveal landscape-level differences in marten gut microbiomes relative to disturbance. A future study with greater sample size (i.e., more individuals) conducted during non-winter months would be valuable both to gauge the degree of gut microbial variation within this carnivore species, and to more thoroughly assess the impact of resource quality and the extent of human disturbance on marten gut microbiomes.

In summary, we provide the first characterization of marten gut microbiomes in a wild population. Importantly, we determined that fecal samples obtained via live-trapping and from harvest were comparable as indicated by indistinguishable gut microbial community composition and distribution patterns based on Chao1 and Faith’s PD. As such, where legal harvest occurs, opportunities exist to engage fur harvesters as citizen scientists to expand marten sample sizes across the harvest season (e.g., winter). Partnerships between fur harvesters and ecologists engaged in research that requires live-trapping marten could expand fecal sampling across the marten geographic range. Broadening the spatial and temporal scales of study would facilitate the assessment of seasonal shifts in marten gut microbiomes–particularly during times of year when marten consume a more omnivorous diet and may rely on a more specialized gut bacterial community to derive nutrients from vegetation (e.g., raspberries). Further, comparing gut microbiomes across species with and without ceca, and including species that consume a variety of diets and occupy diverse land cover types, would be particularly helpful for quantifying the effects of differences in gut morphology, dietary diversity and habitat quality on gut microbial diversity and community structure. Our data underscore the utility of the gut microbiome as a tool for wildlife management–both for monitoring the health of populations and for appreciating the importance and ramifications of gut microbial diversity and community composition across species with diverse ecological roles and requirements.
